# Vitamin D Metabolites: Analytical Challenges and Clinical Relevance

**DOI:** 10.1007/s00223-022-00961-5

**Published:** 2022-03-03

**Authors:** N. Alonso, S. Zelzer, G. Eibinger, M. Herrmann

**Affiliations:** Clinical Institute of Medical and Chemical Laboratory Diagnostics, Medical University of Graz, Graz, Austria

**Keywords:** Vitamin D, Vitamin D metabolites, Chronic kidney disease, Bone health

## Abstract

Recent research activities have provided new insights in vitamin D metabolism in various conditions. Furthermore, substantial progress has been made in the analysis of vitamin D metabolites and related biomarkers, such as vitamin D binding protein. Liquid chromatography tandem mass spectrometric (LC–MS/MS) methods are capable of accurately measuring multiple vitamin D metabolites in parallel. Nevertheless, only 25(OH)D and the biologically active form 1,25(OH)2D are routinely measured in clinical practice. While 25(OH)D remains the analyte of choice for the diagnosis of vitamin D deficiency, 1,25(OH)2D is only recommended in a few conditions with a dysregulated D metabolism. 24,25(OH)2D, free and bioavailable 25(OH)D, and the vitamin D metabolite ratio (VMR) have shown promising results, but technical pitfalls in their quantification, limited clinical data and the lack of reference values, impede their use in clinical practice. LC–MS/MS is the preferred method for the measurement of all vitamin D related analytes as it offers high sensitivity and specificity. In particular, 25(OH)D and 24,25(OH)2D can accurately be measured with this technology. When interpreted together, they seem to provide a functional measure of vitamin D metabolism beyond the analysis of 25(OH)D alone. The determination of VDBP, free and bioavailable 25(OH)D is compromised by unresolved analytical issues, lacking reference intervals and insufficient clinical data. Therefore, future research activities should focus on analytical standardization and exploration of their clinical value. This review provides an overview on established and new vitamin D related biomarkers including their pathophysiological role, preanalytical and analytical aspects, expected values, indications and influencing conditions.

## Introduction

Vitamin D deficiency has developed into a global health issue that affects males and females of all age groups. The lack of sun exposure and a limited availability of vitamin D from natural food sources are the main causes of vitamin D deficiency in modern societies. Other risk factors that can cause or exacerbate vitamin D deficiency are dark skin pigmentation, pregnancy, chronic inflammatory bowel disease with malabsorption, obesity, and advanced age [1]. Acknowledging the global dimension of vitamin D deficiency has sparked an exponential rise in vitamin D testing [2–4] and research activities that have resulted in new insights in vitamin D metabolism in various conditions [5–10]. Today, it is well established that vitamin D has pleiotropic effects that go far beyond calcium and phosphate metabolism [6]. For example, vitamin D modulates innate and adaptive immunity, cell growth and differentiation, cardiovascular function and hormonal actions [6, 11]. Furthermore, vitamin D deficiency has been linked to a broad range of clinical conditions including cardiovascular disease, malignancies, autoimmune diseases, neuropsychiatric diseases and endocrinopathies [6, 11]. Substantial progress has also been made in the analysis of vitamin D metabolites and related biomarkers, such as vitamin D binding protein (VDBP). Mass spectrometric methods are capable of measuring multiple vitamin D metabolites in parallel [12–14]. Recently, Jenkinson et al. have developed a method that can measure 13 different vitamin D metabolites, including 25(OH)D, 24,25(OH)2D and 1,25(OH)2D [13]. While this method is rather complex, the parallel measurement of 25(OH)D and 24,25(OH)2D can easily be performed with liquid-chromatography tandem mass spectrometry using standard instrumentation. Several groups have suggested that the parallel measurement of these two metabolites provides valuable information beyond 25(OH)D alone [15–18]. This development has recently been recognized by DEQAS, an external quality assessment program for vitamin D. However, clinical guidelines still unanimously recommend assessing vitamin D status by the measurement of 25(OH)D. Furthermore, 1,25(OH)2D is only indicated in a very few clinical conditions, such as severe chronic kidney disease, hereditary phosphate-losing disorders, oncogenic osteomalacia, pseudovitamin D-deficiency rickets, vitamin D-resistant rickets, as well as chronic granuloma forming disorders such as sarcoidosis and some lymphomas [19, 20]. However, recent evidence from experimental and clinical studies merits a closer look into the utility and the analytical standards of traditional and emerging biomarkers of vitamin D metabolism. The aim of this review is to provide structured information on 25(OH)D, 1,25(OH)2D, 24,25(OH)2D, VDBP and the derived parameters vitamin D metabolite ratio (VMR), free (free-25(OH)D) and bioavailable (bio-25(OH)D) 25(OH)D. Particular focus will be put on the clinical utility, preanalytical, analytical and postanalytical aspects, and interfering factors.

## Vitamin D Metabolism

Vitamin D is not a single compound, but refers to a group of over 50 metabolites, which are derived from cholesterol via a complex cascade of enzymatic and non-enzymatic reactions [19]. Chemically, they are secosteroids that are characterized by a broken bond in one of the steroid rings. The individual metabolites vary greatly in their plasma concentrations and biological activities [21]. Cholecalciferol (vitaminD_3_) and ergocalciferol (vitamin D_2_) are the two main forms of vitamin D. The difference between the two is an additional double bond between carbons 22 and 23 and a methyl group on carbon 24 in the side chain of vitamin D_2_ [22]. Vitamin D_3_ is primarily synthesised in human skin under the effect of sunlight. In contrast, vitamin D_2_ is exclusively obtained from exogenous sources. Many food items, such as fatty fish, liver oil, and egg yolk, contain vitamin D_2_ and D_3_ so that the diet contributes 10–20% to the vitamin D supply of humans [23–25]. Higher amounts of vitamin D can be obtained from fortified foods, such as milk and margarine, or vitamin supplements [26].

Circulating vitamin D_2_ and D_3_ are activated by two hydroxylation reactions that occur in the liver and the kidneys. The hepatic cytochromes P450 CYP2R1 (microsomal) and CYP27A1 (mitochondrial) hydroxylate vitamin D_2_ and D_3_ at carbon 25 resulting in the production of 25(OH)D, the most abundant vitamin D metabolite in blood, which is still inactive. Renal CYP27B1 attaches the second hydroxygroup in position 1 forming active 1,25-(OH)_2_D [27]. In addition to the kidneys, CYP27B1 is also expressed in many other cell types so that 1,25-(OH)_2_D can be produced by most extra-renal tissues, where it has primarily autocrine or paracrine function. However, this extra-renal 1,25-(OH)_2_D synthesis contributes little to the circulating concentration of this metabolite [28]. Vitamin D degradation is predominantly driven by CYP24A1, which metabolises 25(OH)D to 24,25-dihydroxy-vitamin D [24,25(OH_2_D)] and 1,25(OH)_2_D to 1,24,25-trihydroxy-vitamin D [1,24,25(OH)_3_D][27]. In the circulation, all vitamin D metabolites are bound to vitamin D binding protein (DBP), albumin and lipoproteins.

The C3 hydroxyl group of 25(OH)D_2_ and 25(OH)D_3_ can be converted to beta orientation resulting in the formation of 3-epi-25(OH)D_3_, 3-epi-25(OH)D_2_. It is believed that this irreversible modification is catalysed by the enzyme 3-epimerase (C-3 epimerisation pathway), which also epimerizes 1α,25(OH)_2_D_3_ and 1 α,25(OH)_2_D_2_. Similar to the non-epimerized metabolites, also 3-epi-25(OH)D_3_, and 3-epi-25(OH)D_2_ can be further hydroxylized by 1α-hydroxylase forming 3-epi-1 α,25(OH)_2_D_3_ and 3-epi-1 α,25(OH)_2_D_2_. So far, 3-epimerase has been identified in the endoplasmatic reticulum of liver, bone and skin [29, 30]. In serum from adult humans, the concentration of 3-epi-25(OH)D_3_ varies between 1 and 25% [31]. Children appear to have markedly higher proportion of 3-epi-25(OH)D_3_ reaching up to 60% [32]. With the methods that are commonly used in medical laboratories, the concentrations of all other 3-epimers are too low to be detected. In a study from Shah et al. the mean concentrations of 3-epi-25(OH)D_3_, and 3-epi-25(OH)D_2_ were 6.1 nmol/L and 1.1 nmol/L, respectively. The role of vitamin D 3-epimers in health and disease is still a matter of debate as most of our knowledge is derived from in vitro studies. Apparently, 3-epi-25(OH)D has low affinity for VDBP (36–46% compared with 25(OH)D) and VDR (2–3%), which explains at least partly the low serum concentration. At present it is believed that 3-epi-25(OH)D does not adequately reflect vitamin D status and thus its separate measurement is not recommended. Existing knowledge indicates that 1 α,25(OH)_2_D_3_ harbours a lower biological activity and has a markedly lower affinity to the VDR than 1α,25(OH)_2_D_3_. Also, its ability to stimulate intestinal calcium absorption is significantly reduced [33–35]. PTH suppression, however, was detected at similar rates as 1,25(OH)_2_D_3_ (reviewed in [32]).

## 25(OH)D

### Role of the Marker

The inactive prohormone 25(OH)D represents the main reservoir and transport form of vitamin D. The generation of 25(OH)D from pro-vitamin D_3_ is catalyzed by CYP27A, a 25-hydroxylase encoded by the *CYP2R1* gene [36]. As the most abundant vitamin D metabolite in blood, 25(OH)D includes the two forms 25(OH) D_2_ and 25(OH)D_3_ [37]. Hydroxylation of 25(OH)D in position 1 is mediated by CYP27B1 and results in the formation of active 1,25(OH)_2_D. However, this alpha-hydroxylation occurs on demand, primarily in the kidneys, under the control of parathyroid hormone (PTH). Consequently, the serum concentration of 1,25(OH)_2_D is kept within the reference range over a wide concentration range of 25(OH)D and does not reflect vitamin D stores. In fact, individuals with a low serum 25(OH)D concentration often have a high-normal or raised 1,25(OH)2D concentration due to a compensatory induction of CYP27B1. Therefore, current guidelines unanimously recommend 25(OH)D as the preferred indicator of the body’s vitamin D stores.

People with dark skin have 30–40% lower 25(OH)D serum concentrations than Caucasians but comparable or higher bone mineral density (BMD) and lower fracture risk [38]. Furthermore, lower serum 25(OH)D concentrations in blacks are not associated with higher PTH concentrations, lower BMD or increased fracture risk [39–42]. This conundrum is at least partly explained by a high prevalence (> 90%) of the GC1F haplotype of VDBP (rs7041-T/rs4588-C) in blacks, whereas the GC1S haplotype of VDBP (rs7041-G/rs4588-C) is dominant in Caucasians [43]. While carriers of the GC1F and the GC1S haplotypes show comparable VDBP concentrations [43] they differ in their affinity for most vitamin D species, which contributes to the different 25(OH)D concentrations. However, the availability of free vitamin D for metabolism is not affected. Berg et al. have shown that blacks and whites with comparable serum PTH concentrations also have a comparable VMR despite substantially lower 25(OH)D levels [44]. Other factors that contribute to lower 25(OH)D concentrations in blacks include adiposity and skin pigmentation. A recent report from an international expert panel stated that no one factor alone could fully explain the vitamin D paradox in Black Americans. In addition, blacks have no skeletal benefits from high doses of vitamin D supplementation [45].

### Pre-analytical Considerations

The concentration of 25(OH)D can be assessed in both, serum and plasma, with similar results [46]. A low biological variability [47, 48] in combination with rather high concentrations in the nM range and a long in vitro half-life make 25(OH)D a robust biomarker that can reliably be measured by clinical laboratories [14, 19, 20]. A recent study from Denmark has shown that the in vivo half life depends on the individual 25(OH)D concentration and the vitamin D receptor (*VDR*) gene polymorphism rs2228570 [49]. In individuals with a 25(OH)D start concentration between 68 and 213 nmol/L, mean half-life was 89 days, whereas lower concentrations were associated with a half-life between 149 and 199 days. The longer half-live at lower 25(OH)D concentrations may be caused by storage mobilisation, changed catabolism or increased intestinal absorption. In addition, the *VDR* gene polymorphism rs2228570 may explain up to 88% of the observed variation with the genotype GG having a 120 days shorter half-life than the genotype AA/AG. Stability studies have shown that 25(OH)D is stable in whole blood for up to 24 h when kept at room temperature (RT) [50]. Also, storage of serum for up to 72 h at RT or refrigerated at 6 °C has negligible effects on 25(OH)D. Good short term stability of serum 25(OH)D at RT has also been reported by Zelzer S et al. [17]. Long-term stability at − 20 °C and − 80 °C is generally good. In an own study, we were able to demonstrate that changes do not exceed 15% after 2 months [17]. Recently, Cavalier et al. reported only minor changes of 25(OH)D after 5 years of storage at − 80 °C, which are most likely the result of sample concentration rather than degradation [51]. Long-term storage appears to have the least effect on 25(OH)D measured by LC–MS/MS, whereas samples from renal and pregnant patients may show substantial differences when analysed by immunoassay. Importantly, freezing and thawing of plasma and serum samples does not affect the 25(OH)D concentration [52]. Also, centrifugation temperature has no substantial effect on the 25(OH)D concentration [53, 54]. Importantly, serum and plasma samples for the measurement of 25(OH)D should always be stored in the dark. While short term UV-irradiation has little impact on the 25(OH)D concentration, prolonged sun exposure reduces 25(OH)D by more than 50% [54].

### Analytical Considerations

25(OH)D is a challenging analyte due to its strong binding to VDBP and other carriers, the need of measuring 25(OH)D_2_ and 25(OH)D_3_ in an equimolar fashion, the coexistence of multiple chemically related molecules that may cross-react, and common matrix effects, such as heterophilic antibodies or changes in protein composition [19, 20]. Medical laboratories employ different methods for the measurement of 25(OH)D in serum and plasma with automated immunoassays, ELISAs and LC–MS/MS being the most frequently used. Radioimmunoassays (RIA) and high-performance liquid chromatography (HPLC) have been widely used in the past, but due to a broad range of alternatives they play a marginal role in today’s practice. From an analytical point of view, the available methods can be divided in those with and without complete removal of proteins and lipids by strong organic solvents prior to analysis. The first group includes LC–MS/MS, HPLC and, RIAs whereas the second group comprises automated immunoassays. As automated immunoassay-analysers cannot use such organic solvents, they employ alternative strategies to release 25(OH)D from its carriers. However, they have an inferior dissociation efficacy. The strategies that are used by immunoassay manufacturers to separate 25(OH)D from its carriers are optimized for the expected matrix composition. In situations where the matrix is altered, such as in pregnant women, patients with chronic kidney disease or individuals with a polymorphic variant of *VDBP*, these approaches may be less efficient and thus introduce analytical bias. In contrast, the organic solvents used in LC–MS/MS are strong enough to precipitate all proteins and detach all vitamin D metabolites from their carriers [55, 56]. Consequently, automated immunoassays are characterized by a variable analytical performance. In external quality assurance programs, the means of commonly used automated immunoassays show differences of up to 20 nmol/L in the clinically relevant range. Standardization of the 25(OH)D measurement has been a major step forward. With the help of the standard reference materials (SRM) 972 and 972a, developed by the National Institute of Standardization (NIST), and validated reference measurement procedures (RMP) from the University of Gent [57] and the Centre of Disease Control [58] it is possible to accurately determine the 25(OH)D concentration in serum and plasma samples. As these RMPs are not suitable for clinical laboratories, the Vitamin D Standardization and Certification Program (VDSCP) has been developed with the aim to align the results of different methods to this reference system [59]. Today, 38 methods are listed at the CDC homepage as being VDSCP certified (https://www.cdc.gov/labstandards/vdscp_participants.html). However, the main weakness of VDSCP is that certification depends on the mean bias obtained on a standard set of samples, regardless of the scatter that these samples produce around the target values. In other words, a wide scatter can produce a similar mean bias than a narrow scatter, as long as the individual values are distributed equally around the target values. Therefore, Wise et al. have proposed the percentage of samples with a bias ≤ 10% as a better criterion for accuracy [60]. While the different standardization efforts have clearly improved the comparability of 25(OH)D results obtained with automated immunoassays, external quality assurance programs still show unsatisfactory variability. Numerous laboratories have acknowledged the intrinsic variability of automated immunoassays and switched to LC–MS/MS with electrospray ionisation [61] as the gold standard for the quantification of 25(OH)D. This technology is suitable for the analysis of serum, heparin and EDTA samples [62]. It can accurately measure 25(OH)D_2_ and 25(OH)D_3_. Currently, 20% of all participants of the DEQAS program use LC–MS/MS for the measurement of 25(OH)D. This group is consistently well aligned to the target values and copes best with interferences from other vitamin D metabolites and matrix effects.

All LC–MS/MS methods require a more or less complex pre-analytical sample preparation that cleans the sample and extracts vitamin D metabolites through protein precipitation, liquid–liquid extraction and sometimes derivatisation [40]. Regardless of the pre-analytical sample preparation, thoroughly validated LC–MS/MS methods are consistently capable of producing accurate results [17, 62]. For example, Zhang et al. validated a highly specific LC–MS/MS method for the simultaneous quantification of 25(OH)D_2_ and 25(OH)D_3_ in serum and plasma [62]. Using a combination of methanol precipitation and liquid–liquid extraction by heptane, they were able to obtain a 72% recovery with a coefficient of variation (CV) < 7.05%. The results were well aligned to the Vitamin D External Quality Assessment Scheme (DEQAS) LC–MS/MS method mean values, but about 9% higher than the commonly used DiaSorin Liasson assay. However, it should be mentioned that LC–MS/MS methods for the measurement of 25(OH)D also have disadvantages, such as a (semi)manual sample preparation, and the need of expensive instrumentation and experienced staff. In addition, they are not yet suitable for high throughput. Nevertheless, first fully automated solutions are already available.

From the analytical point of view, 3-epi-25(OH)D_3_ co-elutes and has identical mass as 25(OH)D_3_, and they can only be separated by high resolution chromatography [63]. Considering the ongoing controversy about the biological relevance of vitamin D 3-epimers in health and disease the separate measurement of 3-epi-25(OH)D_3_ is not recommended. In addition, most 25(OH)D automated immunoassays do not cross-react with 3-epi-25(OH)D so that they do not represent a relevant issue in clinical practice. Only in children, the higher 3-epi-25(OH)D concentrations may eventually result in a misclassification of vitamin D status, when measured with one of the few 25(OH)D methods that are interfered by C3-epimers, such as protein-binding assay.

### Reference Values

Typically, clinical laboratories provide their results with a reference range that comprises the values between the 2.5th and the 97.5th percentile of a healthy reference cohort. For several reasons, 25(OH)D results should not be interpreted on the basis of such a reference range. The circulating 25(OH)D concentration is subject to pronounced seasonal variation of 20–30% with the highest values in summer and autumn [64]. In 74,235 serum samples from Northern Italy that were collected over a two-year period and analysed by a validated LC–MS/MS method, the 2.5^th^ and 97.5^th^ percentile spanned a rather wide range from 12 to 159 nmol/L. Amongst 2000 healthy Austrian blood donors the traditional reference interval was 28.5–136 nmol/L (unpublished data). However, within the traditional reference interval of 25(OH)D, higher concentrations of this metabolite were associated with improved blood biomarker concentrations of calcium homeostasis and bone turnover. In addition, within the reference range, higher 25(OH)D concentrations are also associated with a lower risk of bone fractures [65–67]. Valcour et al. have shown that PTH continuously decreases with increasing concentrations of 25(OH)D and that there is no threshold above which this relationship plateaus [68]. The same applies to other conditions, such as stroke, cardiovascular disease, diabetes mellitus and autoimmune disease [69–73]. Considering that the risk for numerous clinical conditions substantially varies across the reference interval, clinical cut-offs are recommended for the interpretation of serum 25(OH)D results. Several scientific societies have developed practice guidelines that propose 25(OH)D cut-offs on the basis of clinical risk [74]. Most guidelines distinguish between sufficiency, insufficiency and deficiency [37, 75, 76]. Some guidelines also provide cut-offs from severe deficiency to toxicity as summarised in Table 1 [74, 77–82]. These cut-offs apply to bone health, osteoporosis, fracture risk and general health. The First International conference on Controversies in Vitamin D established that levels of 25(OH)D below 12 ng/mL (30 nmol/L) were associated with an increased risk of rickets and osteomalacia, while concentrations between 50 and 125 nmol/L are sufficient to maintain bone health [83]. However, commonly used 25(OH)D cut-off values apply primarily to Caucasians and Asians. People with dark skin have 30–40% lower 25(OH)D serum concentrations than Caucasians but comparable or higher bone mineral density (BMD) and lower fracture risk [38].

**Table 1:** Vitamin D guidelines for different regions

	Global*	Europe*	USA*	Brazil*	Japan*	Australia*	Gulf Region*
Toxicity	> 250	> 500	> 150				
Sufficiency	50–250	50–225	> 75	75–250	> 75	≥ 50	> 75
Insufficiency	30–75	50–75	50–75	50–74	50–75	30–49	50–75
Deficiency	< 50–25	< 50	< 50	< 50	< 50	< 29	< 50

*All values are reported as nmol/L

### Indications

Measurement of serum 25(OH)D is recommended for the diagnosis of vitamin D deficiency [37, 78, 81]. Individuals at increased risk of vitamin D deficiency are those with fragility fractures, chronic kidney disease, malabsorption, and abnormalities of calcium and phosphate metabolism [84]. Determining serum 25(OH)D is also useful for the differential diagnosis of rickets, osteomalacia, and the monitoring of vitamin D supplementation. Finally, 25(OH)D should be determined in patients with suspected hypervitaminosis D and intoxication. When considering measuring vitamin D in the context of various non-bone related disease, it should be kept in mind that reverse causality and residual confounding cannot be excluded. Rather than being an actionable causal factor for chronic diseases, vitamin D deficiency could simply be the result of an underlying condition that alters vitamin D metabolism, or a lifestyle that is associated with poor health and micronutrient deficiencies. Therefore, the value of measuring 25(OH)D for the assessment and management of non-bone-related diseases is a matter of ongoing debate and not yet recommended.

### Influence of 25(OH)D in Pathological Conditions

Most 25(OH)D in serum is bound to VDBP (85%) and albumin (15%) [85]. Variations in the serum concentrations of these carriers lead to corresponding changes of 25(OH)D. The hepatic 25-hydroxylation of vitamin D was long believed to be unregulated [36]. However, recent studies suggest that both age and metabolism can modulate CYP27A activity. Aged humans and animals are characterized by a lower expression of CYP2R1 than their young counterparts and thus they are less responsive to vitamin D supplementation [86]. As vitamin D can be sequestered in adipose tissue [87], it is not surprising that nutrition and body composition also modulate CYP2R activity [87, 88].

Pregnancy causes a small increase in 25(OH)D which is at least partly driven by an increase in VDBP [89]. However, not all studies found a significant changes of serum 25(OH)D in pregnant women [90, 91]. Figueiredo et al. showed that the serum 25(OH)D concentration during pregnancy is influenced by seasonal effects [92]. Women starting pregnancy during winter showed rising 25(OH)D concentrations, whereas women who fell pregnant in summer exhibited no increase. Interestingly, the placenta possesses the principal enzymes of vitamin D metabolism including CYP2R1, CYP27B1 and CYP24A1 and may thus be involved in the regulation of circulating 25(OH)D levels [93]. Maternal 25(OH)D can crosses the placenta and is the exclusive vitamin D source of the foetus reaching cord blood levels between 75 and100% of the maternal value [94].

Anti-epileptic drugs, such as phenobarbital, carbamazepine, phenytoin and valproate, are known to accelerate vitamin D catabolism and thus lower the serum 25(OH)D concentration [82]. Binding of these drugs to the pregnane X receptor increases 25(OH)D catabolism by an induction of CYP3A4 and CYP24A1. The preganane X receptor is a nuclear receptor that shares homologies with the VDR. In order to mitigate the risk of vitamin D deficiency, reduced BMD and increased fracture risk, patients on long-term treatment with anti-epileptic drugs should receive a prophylactic vitamin D supplementation and have their serum 25(OH)D concentration monitored.

Patients with CKD at stages 3 and 4 usually present deficiency of vitamin D, a marker of poor prognosis [95]. Similarly, 60–80% of pre-dialysis children with CKD also have low levels of 25(OH)D [96]. In addition to low serum 25(OH)D levels, CKD patients are also characterized by elevated phosphate and FGF-23 concentrations. The adverse impact of CKD on calcium and phosphate metabolism is associated with secondary hyperparathyroidism. However, the management of vitamin D status in nephrology is still a matter of ongoing debate. In any case, CKD patients with 25(OH)D deficiency should always be treated, regardless of PTH [95]. Vitamin D deficiency in Stages 1 to 3a follow general population recommendations. Later stages are treated with calcitriol or vitamin D analogues, depending on patient characteristics [97].

Obese and bariatric surgery patients frequently present with vitamin D deficiency. Fat soluble vitamin D can be sequestered in adipose tissue resulting in reduced circulating 25(OH)D concentrations. Instead, bariatric surgery compromises the absorption of dietary vitamin D [98, 99]. To prevent adverse skeletal effects, vitamin D deficiency should be diagnosed and corrected prior to bariatric surgery [100]. However, it may be difficult to normalize 25(OH)D and PTH in these patients.

Vitamin D levels have also been investigated in cancer and cardiovascular disease (CVD). Observational studies identified an increased CVD mortality only in vitamin D deficient subjects, but not individuals with normal 25(OH)D serum concentrations. Large randomized intervention studies, such as the VITAL study (25,000 individuals aged 50 years or older treated with 2000 IU/day of vitamin D and 1 g/day of omega-3 fatty acid for over 5 years) [101] and the VIDA study (5000 individuals over 50 years of age treated with an initial dose of 200,000 IU and then 100,000 IU/month of vitamin D for 3.3 years) [102] failed to show any beneficial effect of vitamin D supplementation on the incidence of invasive cancer or the appearance of CVD. The lacking effect of vitamin D supplementation on CVD and cancer incidence is further supported by meta-analyses that pooled randomised clinical trials (reviewed in [103]). However, in line with the observational studies mentioned before, a reduced risk was found in vitamin D deficient subjects. Although existing intervention studies failed to show reductions in CVD and cancer risk, the results may be biased due to some pitfalls, such as the lack of appropriate controls that are completely naive to oral vitamin D intake via the food or de-novo synthesis by the skin [103]. Based on existing observational and intervention studies, measurement of serum 25(OH) is primarily useful to identify vitamin D deficient individuals, where supplementation appears to have beneficial effects on CVD and cancer risk. It should be kept in mind that the interpretation of 25(OH)D results in CVD and cancer patients could be complicated by residual confounding and reverse causality, as vitamin D insufficiency could be the result of poor health and disease specific alterations of vitamin D metabolism [104]. Therefore, the quantification of additional vitamin D metabolites could aid the assessment of vitamin D status in these patients.

Due to its immune-modulatory function, vitamin D has also been investigated in COVID-19 patients. Observational studies have shown that individuals with vitamin D deficiency have an increased risk to develop severe COVID-19 (as reviewed in [105]). However, a retrospective study by Mangge et al. failed to find any association between 25(OH)D_3_, 25(OH)D_2_, 24,25(OH)_2_D_3_ and 25,26(OH)_2_D_3_) and clinical outcome measures, such as recovery rate, death or the need of respiratory support [106]. In the absence of COVID-19-specific prevention studies, some information can be derived from vitamin D supplementation studies that measured the incidence of other infectious respiratory diseases, especially in children. Again, while these studies did not show significant effects in individuals with adequate 25(OH)D concentrations, those with vitamin D deficiency benefitted from vitamin D supplementation. As these studies used primarily historical 25(OH)D analyses, their results may be biased. Based on the limited evidence available, the role of vitamin D in SARS-CoV2-infected patients remains controversial. However, there is some support for a correction of vitamin D deficiency in SARS-CoV2-infected patients to prevent severe disease and adverse outcome.

## Measurement of 1,25(OH)2D

### Role of the Marker

1,25(OH)_2_D is the active form of vitamin D that is produced through 1α-hydroxylation of 25(OH)D. This hydroxylation step is catalysed by CYP27B1 (1α-hydroxylase) and occurs mainly in the kidneys. Consequently, the circulating 1,25(OH)_2_D concentration primarily reflects renal production. Meanwhile, it is well established that many other cells and tissues, such as monocytes, macrophages, dendritic cells, osteoblasts, and keratinocytes, also express 1α-hydroxylase and thus are cable of producing 1,25(OH)_2_D locally [107]. However, 1,25(OH)_2_D that is produced by extra-renal tissues has mainly auto- and paracrine functions and adds little to the serum level [108, 109]. The biological activity of 1,25(OH)_2_D is mediated by binding to the VDR [5], a nuclear receptor that binds specific DNA sequences located at different genomic regions (vitamin D response elements), to subsequently modulate the expression of the respective target genes [110].

With concentrations in the lower pmol/L range, 1,25(OH)_2_D circulates at much lower levels than 25(OH)D. The half-life of 1,25(OH)_2_D is only 4–6 h, which makes serum levels rather volatile. Both metabolites have similar physical and chemical properties [111–113], but unlike 25(OH)D, 1,25(OH)_2_D does not reflect the storage levels of vitamin D in the body [114]. Supplementation of vitamin D increases serum 25(OH)D, but has no measurable effect on serum 1,25(OH)_2_D [115, 116]. Some studies suggest that there is no relationship between both metabolites [117, 118]. In fact, it appears that 1,25(OH)_2_D is produced on demand depending on the cellular need, whereas 25(OH)D reflects the stores that are available for utilization.

The main role of 1,25(OH)_2_D is regulating blood calcium levels through intestinal absorption, renal reabsorption and release from bone stores [119]. When the calcium intake is low, PTH increases the synthesis of 1,25(OH)_2_D, which subsequently activates the transport of calcium from the intestinal lumen to the blood by upregulating *TRPV6* and other genes, such as *S100G*, *ATP2B1*, and *CLDN2*, in the intestine [120]. When calcium intake increases, PTH secretion is reduced leading to a decrease in 1,25(OH)_2_D and activation of a paracellular route, a non-saturable and passive transport through the intercellular space between adjacent cells, which is also modulated by 1,25(OH)_2_D (reviewed in [121]). Therefore, 1,25(OH)_2_D is a key regulator of bone mineralisation [122]. The release of calcium from bone is accompanied by an increased secretion of fibroblast growth factor 23 (FGF23) by osteocytes, which, upon binding to Klotho, inhibits 1-alpha hydroxylase and ultimately reduces the renal synthesis of 1,25(OH)_2_D [123].

In addition to the regulation of calcium homeostasis, 1,25(OH)_2_D also supports bone formation by increasing the osteoblastic expression of alkaline phosphatase, osteocalcin and ostepontin [124]. Furthermore, it stimulates bone resorption by increasing osteoclastogenesis and osteoclast activity [125]. 1,25(OH)_2_D is also involved in the regulation of cell proliferation, differentiation and apoptosis [126]. Experimental evidence from 1α-hydrolase knockout mice suggests that low levels of 1,25(OH)_2_D increase age-related bone loss by activating senescence pathways, like p16/p19 [127]. An adequate supply with 1,25(OH)_2_D could thus preserve bone health by activating an anti-ageing mechanism [128].

In addition to its role in bone, 1,25(OH)_2_D also attenuates the inflammatory response of monocytes and macrophages and increases the expression of anti-inflammatory compounds like IL-10. In contrast, the secretion of pro-inflammatory cytokines like IL-1b, IL-6, TNFα, RANKL and COX2 is reduced by 1,25(OH)_2_D [129]. These immunomodulatory effects may explain the 1,25(OH)_2_D-mediated risk reduction of autoimmune diseases like multiple sclerosis [130]. Blood pressure, insulin secretion and insulin sensitivity are also modified by calcitriol [131–133]. These associations could, at least partially, be due to the regulatory role of 1,25(OH)_2_D in calcium homeostasis. Also, the renin-angiotensin axis is influenced by 1,25(OH)_2_D, which may explain the association with hypertension (reviewed in [134]).

Recent evidence suggests that 1,25(OH)_2_D is also linked to the gut microbiome. High serum concentrations of 1,25(OH)_2_D, but not 25(OH)D, are strongly correlated with alpha and beta diversity, especially with butyrate-producing bacteria, like Firmicutes. Based on these results, it has been hypothesized that butyrate-producing bacteria could stimulate the local production of 1,25(OH)_2_D by dendritic cells in the colon [135]. In this context, a previous animal model lacking *VDR* and *CYP27B1* also showed impaired levels of 1,25(OH)_2_D and reduced number of Firmicutes [136].

### Pre-analytical Considerations

1,25(OH)_2_D has similar physical and chemical properties as 25(OH)D; however, it is more hydrophilic, has shorter half-life (around 6 h), and it is found in blood at very low concentrations (in the range of picomol/L) [137]. Altogether, it presents similar challenges as 25(OH)D for analytical detection, but requires a substantially higher sensitivity [46]. Because of these characteristics, results are more volatile.

### Analytical Considerations

Measurement of 1,25(OH)_2_D in serum or plasma is considerably more challenging than 25(OH)D due to is very low concentration. In addition, a standard reference material and a reference measurement procedure are lacking. The first assay for the measurement of 1,25(OH)_2_D was a radioreceptor binding assay, where 1,25(OH)_2_D in the sample displaced tritiated hormone from a cytosol-chromatin receptor preparation isolated from chick small intestine [138]. With this assay, Brumbaugh et al. were able to determine a human plasma concentration of 60 pg/ml in patients with renal disease. In the following years competitive protein binding assays [139], RIAs [140], enzyme immune assays (EIAs) [141], HPLC [142–144], gas chromatography-mass spectrometry (GC–MS) [145] and LC–MS/MS [146] methods were developed. A comprehensive overview of available methods and their characteristics is provided by several review articles [147, 148]. Until recently, commercial RIAs were widely used to determine 1,25(OH)_2_D in clinical laboratories. With the development of fully automated immunoassays [141, 149] this picture has changed. In 2017, 75% of the participants in the DEQAS program used automated immunoassays, 15% manual immunoassays and 9% LC–MS/MS. However, the comparability of automated immunoassays with LC–MS/MS methods is not ideal [150]. Even within the individual method groups substantial variability exists. In this context, different calibration procedures are a common source of variability [151]. LC–MS/MS methods adopt different strategies to cope with the very low analyte concentration. Vitamin D metabolite profiling, and in particular measurement of 1,25(OH)_2_D, typically requires a dual column system, where the first column serves for analyte enrichment and the second one for separation. Other methods include a preanalytical immunopurification step [149, 152, 153] whereas others do not [154]. The antibody used for immunopurification is supposed to enrich the target analyte 1α-25-dihydroxyvitamin D and thus reduces interferences from isobaric compounds, such as 1β-25-dihydroxyvitamin D [84]. Another way to improve analytical sensitivity is the derivatization of 1,25(OH)_2_D [46]. PTAD (4-phenyl-1,2,4-triazoline-3,5-dione) is the most frequently used derivatization agent for this purpose. Also the ionization mode impacts the analytical performance of LC–MS/MS methods [63]. While derivatization methods typically use electrospray ionization (ESI), atmospheric-pressure chemical ionisation (APCI) is a valid alternative that achieves good results without the need of derivatization [63]. However, in the absence of standardization it is not possible to conclude that one method is more accurate than another.

### Reference Values

In contrast to 25(OH)D, where results are interpreted on the basis of clinical cut-offs, method specific reference intervals are recommended for 1,25(OH)_2_D. In healthy adults, the reference interval for the IDS RIA is 43–168 pmol/L, whereas the automated IDS iSYS immunoassay has a reference interval of 63–228 pmol/L [141]. A different reference interval of 59 -159 pmol/L has been reported for LC–MS/MS. However, these methods typically measure only 1,25(OH)_2_D_3_. For a separate quantitation of 1,25(OH)_2_D_2_ most methods are not sensitive enough as the circulating level has been reported to be < 17 pmol/L [155]. In addition, immunoassays may be interfered by chemically related vitamin D metabolites that cross-react.

Blood levels of 1,25(OH)_2_D in children are higher than in adults. The following paediatric reference intervals have been established by Higgins et al. on the CALIPER cohort using the DiaSorin Liason XL method: 77—471 pmol/L between 0 and 1 year, 113–363 pmol/L between 1 and 3 years, and 108 – 246 pmol/L for children older than 3 years. The CALIPER cohort includes 377 Canadian children and adolescents [156]. For other methods, paediatric reference intervals have not been reported.

### Indications

To date, the clinical utility of 1,25(OH)_2_D is insufficiently understood, which limits its clinical use. Serum levels of 1,25(OH)_2_D have little or no relationship to vitamin D stores but rather are regulated by PTH. Measurement of 1,25(OH)_2_D is helpful in the investigation of patients with unexplained hypercalcaemia, sarcoidosis, granulomatous disorders, pseudo-vitamin D deficiency, rickets, tumour-induced osteomalacia, hyperparatiroidism, and CYP24A1 deficiency [20]. Moreover, measurement of this vitamin D metabolite can be helpful to differentiate between FGF23-dependent and –independent phosphopenic rickets [157]. Levels of 1,25(OH)_2_D are usually low in CKD, but its measurement has only been recommended when patients present severe and progressive hyperparathyroidism [158].

### Influence of 1,25(OH)2D in Pathological Conditions

Low serum levels of 1,25(OH)_2_D are observed in CKD and hypoparathyroidism [159]. In contrast, elevated serum concentrations occur in patients with sarcoidosis and tuberculosis. Antimycotic drugs, such as ketoconazole reduce the circulating 1,25(OH)_2_D concentration and have successfully been used to treat tuberculosis-associated hypercalcaemia [160, 161]. Pregnancy increases the serum 1,25(OH)_2_D concentration due to an induction of renal 1α-hydroxylase activity [162]. In addition, 1α-hydroxylase and the VDR are both expressed in the placenta. However, the function of placental 1,25(OH)_2_D synthesis is poorly understood. Serum 1,25(OH)_2_D levels are also higher in African individuals of all age-groups [163, 164], which may be due to increased PTH levels in this population [165]. However, the differences in the metabolism of vitamin, calcium and phosphate are insufficiently understood. Mutations of genes involved in 1,25(OH)_2_D metabolism also influence circulating 1,25(OH)_2_D levels (reviewed in [121]). These mutations cause rare hereditary metabolic bone conditions, like hereditary vitamin D-resistant rickets (*VDR*), vitamin-D-dependent rickets type 1A (*CYP27B1*), type 1B (*CYP2R1*) or idiopathic infantile hypercalcemia (*CYP24A1*). Mutations of the cell surface metalloproteinase *PHEX* cause X-linked hypophosphataemia (XLH), which is characterized by low-normal 1,25(OH)_2_D concentrations [166].

## Measurement of 24,25(OH)_2_D and Calculation of the 24,25(OH)_2_D/25(OH)D Ratio

### Clinical Utility

24,25(OH)_2_D is the principal catabolite of 25(OH)D that is formed through the action of CYP24A1 when sufficient amounts of 25(OH)D are available. Under physiological conditions the serum concentration of 24,25(OH)_2_D ranges at approximately 10% of the 25(OH)D concentration. Normally, 25(OH)D and 24,25(OH)_2_ are highly correlated [167, 168]. Therefore, the isolated measurement of 24,25(OH)_2_ does not provide superior information when compared to 25(OH)D. However, the simultaneous determination of both metabolites has been found useful to identify patients with reduced CYP24A1 activity. Affected individuals are characterized by a reduced ratio between 24,25(OH)_2_D and 25(OH)D [17]. This ratio is also known as vitamin D metabolite ratio (VMR). *CYP24A1* loss-of-function mutations cause infantile hypercalcemia (IIH) and represent a genetic risk factor for serious adverse effects in response to vitamin D supplementation [169]. In hypercalcaemic patients, the parallel measurement of both vitamin D metabolites and calculation of the VMR allows distinguishing CYP24A1 deficiency from other causes of hypercalcaemia, such as vitamin D intoxication, granulomatous disease, Williams-Beuren syndrome or mutations of the *SLC34A1* gene [18, 170]. Therefore, 24,25(OH)_2_D and 25(OH)D can be helpful in guiding genetic testing in patients with inherited hypercalcaemia.

In addition to the rather rare conditions mentioned before, Cavalier et al. have proposed the simultaneous quantitation of 24,25(OH)_2_D and 25(OH)D as a dynamic measure of vitamin D metabolism that aids the identification of individuals with functional vitamin D deficiency [15]. In a cohort of 1200 infants, children, adolescent and young adults, the vast majority of individuals with low 25(OH)D had 24,25(OH)_2_D concentrations below the lower level of quantitation. With increasing 25(OH)D levels, the percentage of individuals such a low 24,25(OH)_2_D concentration decreased and was negligible when serum 25(OH)D exceeded 21 ng/ml (52,5 nmol/L). Based on these results, the authors concluded that this cut-off corresponds to biochemical vitamin D sufficiency. However, Cavallier et al. have also shown that different individuals with the same 25(OH)D concentration can have detectable or undetectable 24,25(OH)_2_D levels, suggesting that they are not equally vitamin D sufficient. This observation questions the use of a fixed 25(OH)D cut-off and suggests that an individual evaluation of the vitamin D status based on a simultaneous analysis of 24,25(OH)_2_D and 25(OH)D might be more appropriate. Also, in black individuals, the parallel determination of both metabolites does better reflect the individual vitamin D status than the measurement of 25(OH)D alone. On average, blacks have a 40% lower 25(OH)D serum concentration, which is at least partially due to ethnic differences in the prevalence of common genetic *VDBP* polymorphisms [38]. Recent evidence from a cohort study of elderly community-dwelling individuals has shown that the VMR was associated with the risk of hip fracture whereas 25(OH)D was not [171]. In addition, higher 24,25(OH)_2_D, but not VMR, was associated with a higher bone mineral density (BMD). The management of renal patients may also benefit from the measurement of 24,25(OH)_2_D. In addition to a decreased production of 1,25(OH)_2_D, also vitamin D catabolism is impaired in patients with chronic kidney disease [172]. The Seattle Kidney Study has shown that patients with a 24,25(OH)_2_D serum concentration below the cohort median of 2.4 ng/ml had a significantly lower eGFR and an increased risk of mortality. Moreover, the inverse correlation of 24,25(OH)_2_D with PTH was stronger than that of 25(OH)D or 1,25(OH)2D.

Last, but not least, the VMR has also been proposed as an interesting tool to guide vitamin D supplementation at an individual level [173]. However, in existing vitamin D supplementation studies, neither 24,25(OH)_2_D nor VMR predicted the increase in serum 25(OH)D better than the baseline 25(OH) concentration [118, 174, 175]. In addition, neither 24,25(OH)_2_D nor VMR predicted the biological effect of vitamin D supplementation on bone and mineral metabolism better than 25(OH)D [176]. However, as most participants had detectable 24,25(OH)_2_D in serum, it cannot be excluded that they were vitamin D sufficient, which may have limited the diagnostic potential of this analyte. So far, no study compared the response of vitamin D supplementation in individuals with and without measureable concentrations of 24,25(OH)_2_D, but comparable serum 25(OH)D concentrations.

Current guidelines do not yet recommend the measurement of 24,25(OH)_2_D in clinical practice. However, existing studies suggest that this practice should be changed. The determination of 24,25(OH)_2_D and calculation of the VMR are helpful in the differential diagnosis of patients with hypercalcaemia and seem to improve risk prediction in conditions where vitamin D catabolism is impaired. In addition, the simultaneous measurement of 25(OH)D and 24,25(OH)_2_D may help to individualize the assessment of patients’ vitamin D status. In contrast, neither 24,25(OH)_2_D nor VMR can predict the response to vitamin D supplementation better than 25(OH)D.

### Preanalytical Considerations

24,25(OH)_2_D and 25(OH)D share similar chemical properties. Both compounds are very stable in serum and can thus be kept for up to 2 months at temperatures between + 25 °C and − 80 °C [17, 177]. For long-term storage samples should be frozen. More than 2 freeze-and-thaw cycles should be avoided as the serum 24,25(OH)_2_D concentration decreases by more than 20%. So far, the effect of light exposure and sample matrix on the 24,25(OH)_2_D serum concentration has not been studied systematically. However, it is prudent to protect samples from light exposure. The influence of patient related preanalytical factors, such as food intake, menstrual cycle or sunlight exposure has not been studied systematically. However, it can be expected that in line with 25(OH)D, vitamin D supplementation and sunlight exposure will increase the synthesis of 24,25(OH)_2_D.

### Analytical Considerations

Measurement of 24,25(OH)_2_D is typically performed by LC–MS/MS. Although this technology is considered the gold standard for the measurement of vitamin D and its metabolites, an interlaboratory assay comparison by Wise et al. has shown substantial systemic differences of up to 36% [178]. In order to improve the comparability of 24,25(OH)_2_D results, a standard reference material [179, 180] and a reference method [181] have been developed. In addition, first EQA programs have included this analyte in their portfolio [182]. The efficacy of these measures has been demonstrated by a recent comparison study of two in-house LC–MS/MS methods [16]. Both methods were properly validated and underwent regular proficiency testing. So far, immunoassays or other analytical methods for the measurement of this lowly concentrated analyte have yet not been developed.

### Reference Values

So far, two studies have determined references ranges for the serum concentration of 24,25(OH)_2_D and the VMR using validated LC–MS/MS methods [183, 184]. In 1996, Tang et al. found an average 24,25(OH)_2_D serum concentration of 5.7 ± 3.4 nmol/L and calculated a reference interval (2.5th–97.5th percentile) of 1.1.-13.5 nmol/L in healthy young army recruits (504 females and 1492 males) with a mean age of 23 years [184]. The diagnostic cut-off for vitamin D sufficiency (serum 25(OH)D > 50 nmol/L) was > 4.2 nmol/L. Dirks et al. established a serum 24,25(OH)_2_D reference interval in 92 middle-aged adults that ranged from 0.4 to 8.9 nmol/L [183]. Both studies calculated the VMR as 25(OH)D/24,25(OH)_2_D and reported reference intervals of 10–33 [184] and 7–23 [183], respectively. In line with these results, an earlier study from Ketha et al. reported a VMR reference interval of 7–35, which was obtained in 91 adults with an age range of 28–86 years [185]. It should be mentioned that not all laboratories calculate the VMR in the same way, while some use the formula 25(OH)D/24,25(OH)_2_D, others do it the other way round 24,25(OH)_2_D/25(OH)D, which provides totally different figures. Moreover, the VMR is further limited by the fact the two vastly different numbers are divided by each other and thus small changes in 24,25(OH)_2_D result in a great change in VMR. Furthermore, imprecision of this ratio is affected by the imprecision of the two methods.

## Measurement of Vitamin D Binding Protein (VDBP) and Calculation of Free and Bioavailable 25(OH)D

### Role of the Marker

VDBP is a highly polymorphic protein with over 100 isoforms that is encoded by the *VDBP* gene on chromosome 4q12-q13. Structurally, the protein is closely related to albumin and alpha-fetoprotein [85]. It is mainly produced in the liver, but animal studies suggest that it can also be expressed by other tissues, such as kidney, testis, and adipose tissue [186]. The expression of VDBP is regulated by estrogens, which show higher concentrations during pregnancy and upon oral contraception [85].

As mentioned above, VDBP binds approximately 85% and albumin 15% of all circulating vitamin D metabolites [85]. Less than 0.1% of all 25(OH)D in the plasma of normal individuals is free. Similar to other steroid hormones, it is believed that only the free fraction can enter the cells to bind the cytosolic VDR. The free hormone hypothesis is supported by the observation that lacking VDBP does not necessarily cause functional vitamin D deficiency despite very low serum concentrations of 25(OH)D and 1,25(OH)_2_D [187–189]. In addition to its transport function of vitamin D metabolites, VDBP has also other functions including the renal megalin/cubilin-mediated reuptake of 25(OH)D in the kidneys,the scavenging of actin after muscular damage, neutrophil recruitment, complement 5a-mediated chemotaxis, fatty acid binding and formation of VDBP-Macrophage Activating Factor V (DBP-MAF) [85, 187]. For example, significant amounts of actin are released from damaged cells in response to trauma, sepsis, liver trauma, acute lung injury, preeclampsia, surgery, and burns. Upon polymerization, filamentous F-actin in combination with coagulation factor Va can promote disseminated intravascular coagulation and multiorgan failure. Through binding to VDBP, actin is rapidly cleared by the liver, lungs and spleen, thus preventing polymerization.

Based on the free hormone hypothesis [190] is has been proposed that free 25(OH)D might be a better marker than total 25(OH)D to reflect a patient’s current vitamin D status [38, 191]. For example, patients with liver cirrhosis have a 100% higher median of free 25(OH)D than unaffected controls, despite lower total 25(OH)D concentrations. Interestingly, these patients develop osteoporosis rather than osteomalacia, as it would be expected for vitamin D deficient patients with a low total 25(OH)D concentration [192]. In contrast, pregnant women have lower free 25(OH)D concentrations than non-pregnant controls [193]. Due to existing inconsistencies, the applicability of the free hormone hypothesis to vitamin D is still a matter of ongoing debate [153]. In contrast to other steroid hormones, the active metabolite 1,25(OH)_2_D is feedback regulated and the concentration of free hormone is so low that it may be insufficient for passive diffusion across the cell membrane and effective receptor binding [153]. In addition, it cannot be excluded that the megalin-dependent uptake of the VDBP-25(OH)D complex into cells is limited to the proximal renal tubule. For example, a vitamin D-dependent expression megalin and cubilin has been found in several other epithelia including placenta, parathyroid glands, pneumocytes, and epididymal epithelial cells [93, 194–196].

Considering the much lower affinity of 25(OH)D to albumin (K_a_ = 7 × 10^8^/M) than to VDBP (K_a_ = 6 × 10^5^/M), it is believed that albumin-bound 25(OH)D can easily dissociate from the protein and is thus available for metabolism. This concept has led to the term bioavailable 25(OH)D, which refers to all the circulating 25(OH)D that is not tightly bound to VDBP [197]. Several studies have used bioavailable 25(OH)D rather than free 25(OH)D [38, 198–200].

Free [201] and bioavailable [38] 25(OH)D are calculated from total 25(OH)D, albumin and VDBP:

$${\text{Free}}\,25\left( {{\text{OH}}} \right){\text{D}} = \frac{{{\text{Total}}\,25\left( {{\text{OH}}} \right){\text{D}}}}{{\left( {{\text{k}}_{{{\text{Albumin}}}} {\text{ + k}}_{{{\text{VDBP}}}} } \right)}}$$

$${\text{Total}}\,25\left( {{\text{OH}}} \right){\text{D}} = {\text{free}}\,25\left( {{\text{OH}}} \right){\text{D}} + {\text{albumin-bond}}\,25\left( {{\text{OH}}} \right){\text{D}} + {\text{VDBP-bond}}\,25\left( {{\text{OH}}} \right){\text{D}}$$

$${\text{k}}_{{{\text{Albumin}}}} = 6 \times 10^{5} {\text{/M}}$$

$${\text{k}}_{{{\text{VDBP}}}} = 7 \times 10^{8} {\text{/M}}$$

$${\text{Bioavailable}}\,25\left( {{\text{OH}}} \right){\text{D}} = \left( {{\text{k}}_{{{\text{Alb}}}} \times {\text{Alb}} + 1} \right) \times {\text{free}}\,25\left( {{\text{OH}}} \right){\text{D}}$$

For accuracy, the measurements of 25(OH)D and VDBP are best performed by LC–MS/MS. The polymorphic nature of VDBP results in numerous variants of the protein with Gc1f and Gc1s and Gc2 being most common. These variants are believed to differ in their affinity for 25(OH)D [202, 203]. The lowest k_VDBP_ has been reported for the 1f/1f variant with 3.6 × 10^8^/M, whereas 2/2 has supposedly the highest k_VDBP_ with 11.2 × 10^8^/M. However, different affinities of VDBP variants to 25(OH)D are a matter of ongoing scientific debate [85]. Nevertheless, VDBP haplotypes impact the concentrations of total 25(OH)D, free 25(OH)D, and VDBP. The 2/2 haplotype is associated with the lowest total and free 25(OH)D concentrations. The lowest free percentage was seen with the 1s/1s haplotype and the highest one with the 1f/1f haplotype. Furthermore, the different Gc alleles affect the response to vitamin D supplementation [204]. The clinical significance of these VDBP variants is insufficiently understood. Existing studies do not show differences in fracture rate [44, 205]. However, significant associations with numerous chronic disease, such as type 1 and 2 diabetes, osteoporosis, chronic obstructive lung disease, inflammatory bowel disease, some malignancies and tuberculosis, have been reported [206, 207].

### Preanalytical Considerations

VDBP is relatively stable. Storing serum for 48 h at room temperature or refrigerated introduces only a small positive bias of < 10% [43]. Also, up to 2 freeze–thaw-cycles have no major impact on VDBP. Regarding LC–MS/MS methods, autosampler stability is also acceptable with < 16% loss during 24 h in a 96-well plate. In order to minimize the impact of preanalytical factors, samples should be processed rapidly after collection. The distribution of VDBP variants differs substantially between black and white individuals, which has important analytical implications (see below) and impacts free 25(OH)D levels. Haemolysis and lipaemia have also been reported to interfere with the measurement [43]. In addition, free 25(OH)D is less stable than protein-bound 25(OH)D and thus requires some precautions for direct measurement. Samples are light-sensitive and should be protected from UV-irradiation. Long term storage should be at − 70 °C [208]. To mitigate the impact of preanalytical factors on free 25(OH)D, measurement in fresh samples is preferred.

### Analytical Considerations

The measurement of VDBP is challenging and LC–MS/MS is the preferred method for this analyte due to superior sensitivity and specificity [43]. For example, only LC–MS/MS can detect VDBP isoforms. However, LC–MS/MS analyses require experienced staff and expensive equipment, which are not widely available [208]. Immunoassays have been developed as easy-to-use alternative that allow a relatively high throughput. However, the use of immunoassays for the quantitation of a highly polymorphic protein, such as VDBP, is problematic, especially when the target epitope(s) are located near the polymorphic region [43]. For example, Henderson CM et al. have shown that assays with a monoclonal capture antibody may react differently with common variants, and thus deliver misleading results [43]. With a monoclonal immunoassay, blacks, which carry predominantly the Gc1f variant, have been reported to have substantially lower VDBP concentrations than whites [38, 209]. When using a validated LC–MS/MS method that can distinguish between VDBP variants, no ethnic differences in VDBP concentration were found. Although polyclonal assays are less influenced by isoforms and other genetic variations, they still deliver largely discrepant results when compared to LC–MS/MS with a median bias of + 50% [210]. Such differences have great impact on the calculation of free and bioavailable 25(OH)D [209, 211]. Therefore, free and bioavailable 25(OH)D concentrations should be determined on the basis of accurate VDBP and total 25(OH)D results obtained with validated LC–MS/MS methods.

The direct quantification of free 25(OH)D constitutes an analytical challenge, due to its very low concentration (0.02% to 0.09% of total 25(OH)D concentration) [193]. Even sensitive LC–MS/MS systems are not sufficient to accurately measure such low concentrations. Therefore, most studies calculate the free 25(OH)D concentration from measurements of total 25(OH)D, VDBP and albumin. However, the result is strongly dependent on the accuracy of the methods used for total 25(OH)D and VDBP. A comparison of calculated free 25(OH)D results obtained with four different VDBP immunoassays has shown differences of up to 50%. Direct methods for the measurement of free 25(OH)D have also been developed [212, 213]. Centrifugal ultrafiltration has been described in the 1980s and appears reasonably accurate [212]. However, this method is highly complex and expensive. A recently marketed ELISA from DiaSource showed good agreement with a dialysis-based reference method [213], but low concordance with indirect methods [211, 214]. Amongst 173 healthy women, Deming's regression showed a slope of 0.3 ± 0.03, and a correlation coefficient of 0.6 ± 0.06.

### Expected Values

To date, reliable reference values for free 25(OH)D and VDBP have not yet been established, and several ranges have been proposed. Due to the lack of standardization, reference intervals have to be method-specific. In addition, differences between black and white individuals have been reported for some methods, but not for others. When measuring free 25(OH)D by direct immunoassay in 279 healthy controls with a median age of 36.6 years, 95% of the results ranged between 0.5 and 8.1 pg/ml, with VDBP concentrations measured by a polyclonal immunodiffusion assay between 190.8 and 395.2 µg/ml [193]. Amongst 2085 participants of the Healthy Aging in Neighbourhoods of Diversity across the Life Span cohort, blacks and whites showed mean ± standard error concentrations of 168 ± 3 µg/ml and 337 ± 5 µg/ml, respectively [38]. Bioavailable 25(OH)D-was 2.9 ± 0.1 ng/ml in blacks and 3.1 ± 0.1 ng/ml in whites. However, VDBP was measured by a monoclonal immunoassay that suffers from variable detection of different VDBP variants. In 173 healthy women, Peris et al. reported free 25(OH)D concentrations between 2 and 15 pg/ml using an indirect method whereas directly measured results ranged between 2 and 8 pg/ml [214]. Zeng et al. determined normal free 25(OH)D results on the basis of sufficient total 25(OH)D concentrations and suggested a range between 8.5 and 28.3 pg/ml (equivalent to 30 and 100 ng/ml of total 25(OH)D [74]. In our opinion, this range is rather high. In healthy children, Lopez-Molina et al. calculated that directly measured free 25(OH)D results above 9.8 pmol/L (equal to 3.9 pg/ml) correlate best with a total 25(OH)D concentration > 50 nmol/L [215].

### Indications

Currently, routine measurement of VDBP, free and bioavailable 25(OH)D are not recommended due to unresolved analytical issues and the lack clinical evidence that supports an additional value beyond the measurement of total 25(OH)D. Potential areas of interest for the use of these markers are patients with liver or renal disease, which cause a decreased synthesis or loss of VDBP. Moreover, determining free 25(OH)D could also be useful for pregnant women or women on hormone therapy, because estrogens can upregulate VDBP levels. In the same line, elderly people could also benefit from assessing this marker since VDBP levels can be altered in this group due to a multifactorial effect [216, 217]. However, future clinical studies have to clarify the potential value of VDBP, free and bioavailable 25(OH)D in such patients.

### Influence of VDBP in Pathological Conditions

Different variants of VDBP with different affinities for 25(OH)D influence the measurement of VDBP as well as the determination of free 25(OH)D. As the distribution of VDBP variants changes between blacks and whites, ethnicity needs to be considered when assessing these markers [38]. Based on the method used, differences in VDBP and free 25(OH)D have been found or not [38, 43, 211]. Changes in estrogen levels, such as in pregnancy, alter the expression of VDBP. Between the second and third trimester, VDBP levels increase up to two-fold. However, these variations are not linked to changes in free 25(OH)D, which remains stable or slightly decreases [85, 218, 219].

Critically ill patients with organ dysfunction also show substantial variation in serum VDBP when compared with normal individuals [220, 221]. Liver diseases, especially at late stages, often cause impaired protein synthesis, resulting in decreasing levels of VDBP and albumin. In turn, free 25(OH)D is increased in these patients compared to controls [85]. These changes are most pronounced in cirrhotic patients. Renal disease also decreases VDBP levels, via proteinuria and saturation of the maximal transport capacity of the megalin/cubilin system. Consistently, nephrotic patients present low total and free 25(OH)D levels [222]. However, in dialysis patients VDBP levels are comparable with healthy individuals [218]. Despite normal VDBP levels, immunoassays and LC–MS/MS methods vary in the quantitation of 25(OH)D in dialysis patients. Potential differences may be due to increased urea concentrations that interfere with the release of 25(OH)D from VDBP, the binding equilibrium or the reagents of immunoassays. In contrast, LC–MS/MS methods are not affected from such interferences.

## Conclusion

The existence of multiple vitamin D metabolites with different biological activities and their binding to VDBP and other carriers makes the assessment of vitamin D status a challenging task. Despite promising results for alternative markers, such as 24,25(OH)D, VMR, free and bioavailable 25(OH)D, 25(OH)D remains the preferred analyte for this purpose. The assessment of 25(OH)D is recommended for patients with established metabolic bone disease or individuals with an increased risk of developing these conditions, and the monitoring of vitamin D supplementation. For non-bone-related diseases, the determination of 25(OH)D is not recommended. Despite recent standardization, the accurate measurement of 25(OH)D remains a critical issue in clinical practice. Properly validated LC–MS/MS methods are preferable due to their high sensitivity, specificity, and robustness for interferences from matrix effects. 1,25(OH)_2_D, 24,25(OH) _2_D and the VMR provide functional information on vitamin D metabolism and are thus useful for the investigation of rare enzyme defects (24-hydroxylase deficiency) and some other diseases (e.g. sarcoidosis, tuberculosis). The determination of VDBP, free and bioavailable 25(OH)D is compromised by unresolved analytical issues, the lack of reference intervals and insufficient clinical evidence that justifies their use. Future research should address analytical standardization and explore the clinical value of novel markers of vitamin D metabolism.

## Data Availability

Availability of data and material is not applicable.
